# Practical Real-Time Phase Drift Compensation Scheme for Quantum Communication Systems

**DOI:** 10.3390/e25101408

**Published:** 2023-10-01

**Authors:** Xiaotian Song, Chunsheng Zhang, Dong Pan, Min Wang, Jianxing Guo, Feihao Zhang, Guilu Long

**Affiliations:** 1 Beijing Academy of Quantum Information Sciences, Beijing 100193, China; songxt@baqis.ac.cn (X.S.); zhangcs@baqis.ac.cn (C.Z.); pandong@baqis.ac.cn (D.P.); wangmin@baqis.ac.cn (M.W.); guojx@baqis.ac.cn (J.G.); zhangfh@baqis.ac.cn (F.Z.); 2 State Key Laboratory of Low-Dimensional Quantum Physics and Department of Physics, Tsinghua University, Beijing 100084, China; 3 Frontier Science Center for Quantum Information, Beijing 100084, China; 4 Beijing National Research Center for Information Science and Technology, Beijing 100084, China

**Keywords:** quantum communication, phase drift, real-time phase compensation

## Abstract

Quantum communication systems are susceptible to various perturbations and drifts arising from the operational environment, with phase drift being a crucial challenge. In this paper, we propose an efficient real-time phase drift compensation scheme in which only existing data from the quantum communication process is used to establish a stable closed-loop control subsystem for phase tracking. This scheme ensures the continuous operation of transmission by tracking and compensating for phase drift in the phase-encoding quantum communication system. The experimental results demonstrate the effectiveness and feasibility of the proposed scheme with an average quantum bit error rate of 1.60% and a standard deviation of 0.0583% for 16 h of continuous operation.

## 1. Introduction

Quantum communication [1,2] is a field dedicated to achieving unconditional security between two legitimate parties, namely, Alice and Bob. Over the years, significant advancements have been made in both theoretical and experimental aspects [3,4,5,6,7,8,9,10,11,12,13,14,15,16], garnering considerable attention from diverse disciplines. Notably, the introduction of commercial single-photon level applications of quantum physics [17] has underscored the growing significance of practical quantum communication systems in real-world scenarios. However, it is crucial to acknowledge that real-world application environments are considerably more intricate and diverse compared to controlled laboratory settings. These complexities have the potential to impact system operations and even lead to disruptions.

Coupling with environment will adversely influence the performance of quantum systems [17,18]. Unlike laboratory environments where temperature control is commonly achieved through air conditioning and optical fiber channels are carefully arranged, the field environment lacks specialized environmental control measures and most fiber channels are installed on-site. The impact of field environments on quantum communication systems can be broadly categorized into two main aspects: polarization and phase drift. Polarization issues primarily arise from channel disturbances in installed fiber channels, while phase drift is often caused by interferometer drift. In phase-encoding quantum communication systems, polarization-related challenges can be effectively mitigated through ingenious system architecture design [4,19,20], in which long-term stability can be achieved without the need for polarization compensations. However, addressing phase drift requires careful consideration of operational conditions. Environmental isolation is one possible countermeasure, which reduces the speed of phase drift but increases system complexity [21]. Another popular approach is the active feedback scheme, which has received significant attention. These schemes involve techniques such as using additional reference light for active alignment or performing pre-transmission phase drift parameter scanning [10]. However, such schemes often lead to a reduction in transmission efficiency. Machine learning techniques [22] have also been employed to predict phase drift. However, a substantial amount of training data must be collected beforehand for accurate predictions. More recently, efficient schemes utilizing mismatched data [23] for calibration have emerged, although they still require additional data transmission.

In conclusion, phase drift in field environments poses significant challenges for phase-coding quantum communication systems. While innovative design approaches and active feedback schemes have been explored, further research is needed to enhance transmission efficiency and develop robust solutions for real-world applications. Here, we propose a practical real-time calibration scheme for phase tracking. In our scheme, the system can continuously run without any additional transmission efficiency reduction or information exchange process by a finely designed closed-loop control algorithm, which is validated in our subsequent experiment. Experimental results show that the phase-coding quantum communication system with our phase tracking scheme can be operated stably and continuously for 16 h with an average quantum bit error rate (QBER) of 1.60% and a standard deviation of 0.0583%.

## 2. Methods

In a practical phase coding four-state style quantum communication system [4,20], four phases $0,\frac{\pi }{2},\pi ,\frac{3\pi }{2}$ should be randomly encoded at Alice and Bob’s interferometers, respectively. Considering that most of the commercial phase modulators are based on lithium niobate ($LiNbO_{3}$), which has quite stable half-wave voltage and good modulation linearity under a certain modulation bandwidth [24,25], we assume a linear relationship between the driving voltage and the modulated phase. Additionally, the half-wave voltages at the phase modulators in Alice and Bob’s interferometers are assumed to be constant and denoted as $V_{\pi Ac}$ and $V_{\pi Bc}$, respectively. Therefore, the driving voltages of the four phases can be obtained as

(1) $$\begin{array}{c} \left\{\begin{array}{c} V_{\frac{\pi }{2}A\left(B\right)}=V_{0A(B)}+\frac{1}{2}V_{\pi A(B)c}\\ V_{\pi A(B)}=V_{0A(B)}+V_{\pi A(B)c}\\ V_{\frac{3\pi }{2}A\left(B\right)}=V_{0A(B)}+\frac{3}{2}V_{\pi A(B)c} \end{array}\right., \end{array}$$

where $V_{0A(B)},V_{\frac{\pi }{2}A\left(B\right)},V_{\pi A(B)},V_{\frac{3\pi }{2}A\left(B\right)}$ are the driving voltages of $0,\frac{\pi }{2},\pi ,\frac{3\pi }{2}$ at Alice’s (Bob’s) side, respectively. In addition, $V_{0A(B)}$ can be considered as the reference voltage for the modulation voltages at Alice’s (Bob’s) phase modulator. From Equation (1), we can easily determine that the phase difference between Alice and Bob can be compensated by an additional driving voltage of $V=V_{0A}-V_{0B}$ on the phase modulator. Here, we take the asymmetric Faraday–Sagnac–Michelson interferometer (FSMI)-based quantum communication system [20] for example, and for convenience, we consider the case of interference peak in our analysis, as the non-interference peaks can be filtered out by gated-mode single-photon detector. According to the analysis in Ref. [26], the two outputs of the interferometer at Bob can be described as

(2) $$\begin{array}{c} \begin{array}{c} P_{1\mathrm{out}}=P_{d}+\eta \left(1-v\cos\Delta \varphi \right),\\ P_{2\mathrm{out}}=P_{d}+\eta \left(1+v\cos\Delta \varphi \right), \end{array} \end{array}$$

where $P_{d}$ is the dark count probability of the single photon detector (SPD), η is the quantum efficiency of the photon, which satisfies η≪1, *v* is the interference fringe visibility of the interferometers, and Δφ is the phase difference between Alice and Bob.

With different phase coding definitions of bit 0 and 1 at the two communication parties, the QBER can be given by

(3) $$\begin{array}{c} e=\frac{P_{1\mathrm{out}}}{P_{1\mathrm{out}}+P_{2\mathrm{out}}}=\frac{P_{d}+\eta \left(1-v\cos\Delta \varphi \right)}{2(P_{d}+\eta )} \end{array}$$

or

(4) $$\begin{array}{c} e=\frac{P_{2\mathrm{out}}}{P_{1\mathrm{out}}+P_{2\mathrm{out}}}=\frac{P_{d}+\eta \left(1+v\cos\Delta \varphi \right)}{2(P_{d}+\eta )}. \end{array}$$

It can be clearly seen that the formulas are symmetric and equivalent, so we just consider the first case for example. For simplicity, we define $e_{0}$ as the QBER when Δφ=0:

(5) $$\begin{array}{c} e_{0}=e\left(\Delta \varphi =0\right)=\frac{P_{d}+\eta \left(1-v\right)}{2(P_{d}+\eta )}, \end{array}$$

and Equation (3) can be adjusted to be

(6) $$\begin{array}{c} \begin{array}{c} \begin{array}{cc} \cos\Delta \varphi & =\frac{\left(1-2e\right)\left(P_{d}+\eta \right)}{\eta v}\\ \; & =\frac{\left(1-2e\right)\left(P_{d}+\eta \right)}{\eta (1-\frac{2e_{0}\left(P_{d}+\eta \right)-P_{d}}{\eta })}\\ \; & =\frac{1-2e}{1-2e_{0}} \end{array}. \end{array} \end{array}$$

Further more, the incremental driving voltage that is needed for compensation can be calculated by

(7) $$\begin{array}{c} \begin{array}{c} \Delta V_{i}=\frac{V_{\pi }}{\pi }\Delta \varphi_{i}\\ =\pm \frac{V_{\pi }}{\pi }\arccos\frac{1-2e_{i}}{1-2e_{0}} \end{array}, \end{array}$$

where the subscript i means the parameter of the i-th round of transmission, and after considering the boundary conditions, $e_{i}$ should satisfy with

(8) $$\begin{array}{c} e_{i}=\left\{\begin{array}{cc} e_{0}, & 0\leq e_{i}\leq e_{0}\\ e_{i}, & e_{0}<e_{i}<0.5\\ 0.5, & 0.5\leq e_{i}\leq 1 \end{array}\right.. \end{array}$$

From Equation (7), we can observe that, apart from the value of the driving voltage difference, the direction of voltage adjustment should be considered as well. Here, we define $\Delta e_{i}=e_{i}-e_{i-1}$ to roughly evaluate the correctness of the previous modulation direction, which is represented by $\varepsilon_{i}$, and thus determine whether to change the modulation direction for this time by

(9) $$\begin{array}{c} \varepsilon_{i}=\left\{\begin{array}{cc} \varepsilon_{i-1}, & \Delta e_{i}\leq 0\left|\right|i>0\\ -\varepsilon_{i-1}, & \Delta e_{i}>0\left|\right|i>0\\ 1, & i=0 \end{array}\right.. \end{array}$$

Therefore, Equation (7) can be substituted with the subsequent formula

(10) $$\begin{array}{c} \Delta V_{i}=\varepsilon_{i}\frac{V_{\pi }}{\pi }\arccos\frac{1-2e_{i}}{1-2e_{0}}, \end{array}$$

and the driving voltage value of the next round can be easily obtained by

(11) $$\begin{array}{c} V_{i+1}=V_{i}+\Delta V_{i}. \end{array}$$

In order to achieve a more stable operation for the system, we also introduced the Proportional–Integral–Derivative (PID) algorithm to calibrate the driving voltage with

(12) $$\begin{array}{c} \Delta {V_{i}}^{\prime }=K_{P}\Delta V_{i}+K_{I}\sum_{n=1}^{i}\Delta V_{n}+K_{D}\left(\Delta V_{i}-\Delta V_{i-1}\right), \end{array}$$

where $K_{P},K_{I},K_{D}$ are the coefficients of the PID algorithm, and Equation (7) can be adjust to be

(13) $$\begin{array}{c} V_{i+1}=V_{i}+\Delta {V_{i}}^{\prime }. \end{array}$$

**Figure 1:**
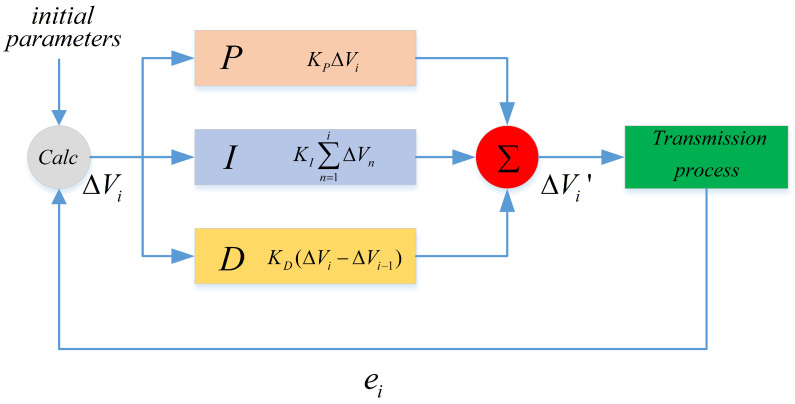
The main flowchart of the real-time phase drift compensation scheme. Calc, the calculation module to calculate the incremental driving voltage $\Delta V_{i}$ using the initial parameters and $e_{i}$; P, I, and D respectively represent the proportional, integral, and derivative components; ∑, sum of the three components.

To conclude, the main flowchart of the proposed scheme is illustrated in Figure 1.

## 3. Experiment and Results

**Figure 2:**
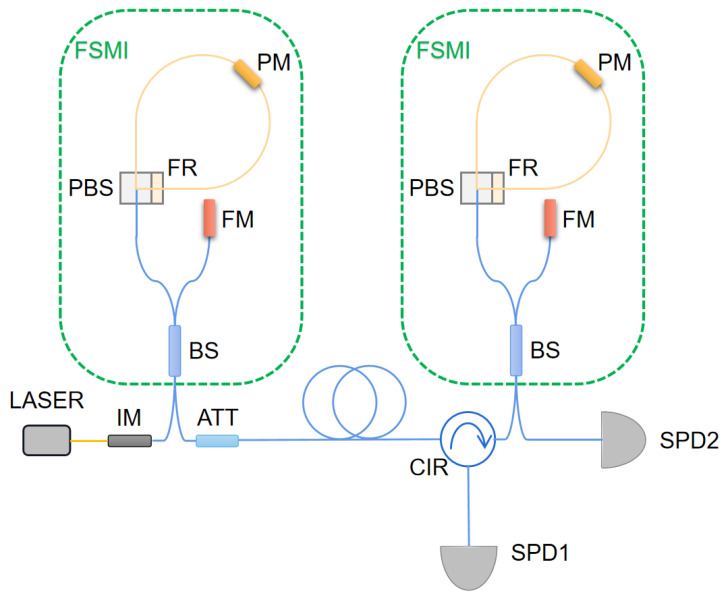
Schematic of the experimental setup. IM, intensity modulator; FSMI, asymmetric Faraday–Sagnac–Michelson interferometer; BS, beam splitter; FM, Faraday mirror; PBS, polarization beam splitter; FR, Faraday rotator; PM, phase modulator; ATT, attenuator; CIR, circulator; SPD, single-photon detector. The yellow lines represent polarization-maintaining fibers (PMFs), and the blue ones are single-mode fibers (SMFs).

**Figure 3:**
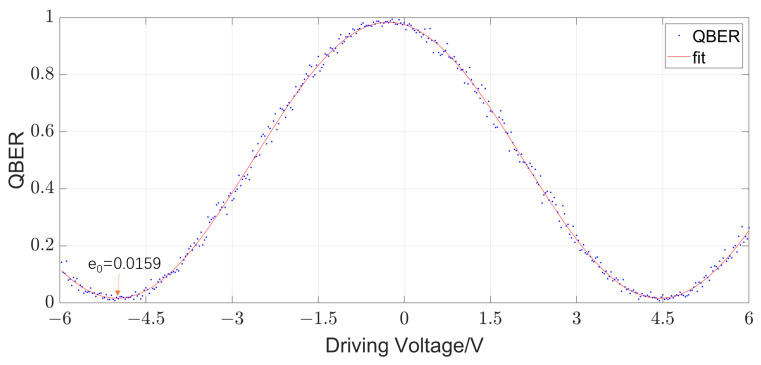
QBER verse Driving Voltage of the phase modulator.

We perform our scheme on an FSMI-based quantum communication system, as schematically shown in Figure 2. A fiber laser produces 1550 nm photon pulses with a width of 50 ps and a repetition frequency of 1.25 GHz; an intensity modulator attenuates the optical pulses to μ=0.6 photons per pulse combined with an attenuator. Benefiting from the sophisticated design of the FSMI, the polarization disturbance in the system need not be considered. The delay of the interferometers is 400 ps and both interferometers are equipped with a phase modulator on their long arms. The phase modulator has three drive voltage interfaces, two of which are RF interfaces for random phase modulation with a repetition frequency of 1.25 GHz and one is a direct current (DC) bias interface for phase compensation. A 12-bit digital-to-analog converter (DAC) is employed for phase tracking, allowing for a total range of 12V and providing a precision of 2.93 mV. The coded pulses are then sent to Bob over a 50 km fiber channel. After transmission, the photons pass through Bob’s FSMI and finally detected by two InGaAs single photon detectors (SPDs), whose average detection efficiency, dark count rate, and afterpulse probability are $20\%,1\times 10^{-6}$, and 1.4%, respectively. It should be noted that, before transmission, we obtain the initialization parameters and $e_{0}=1.59\%$ (shown as Figure 3) through a scanning process.

**Figure 4:**
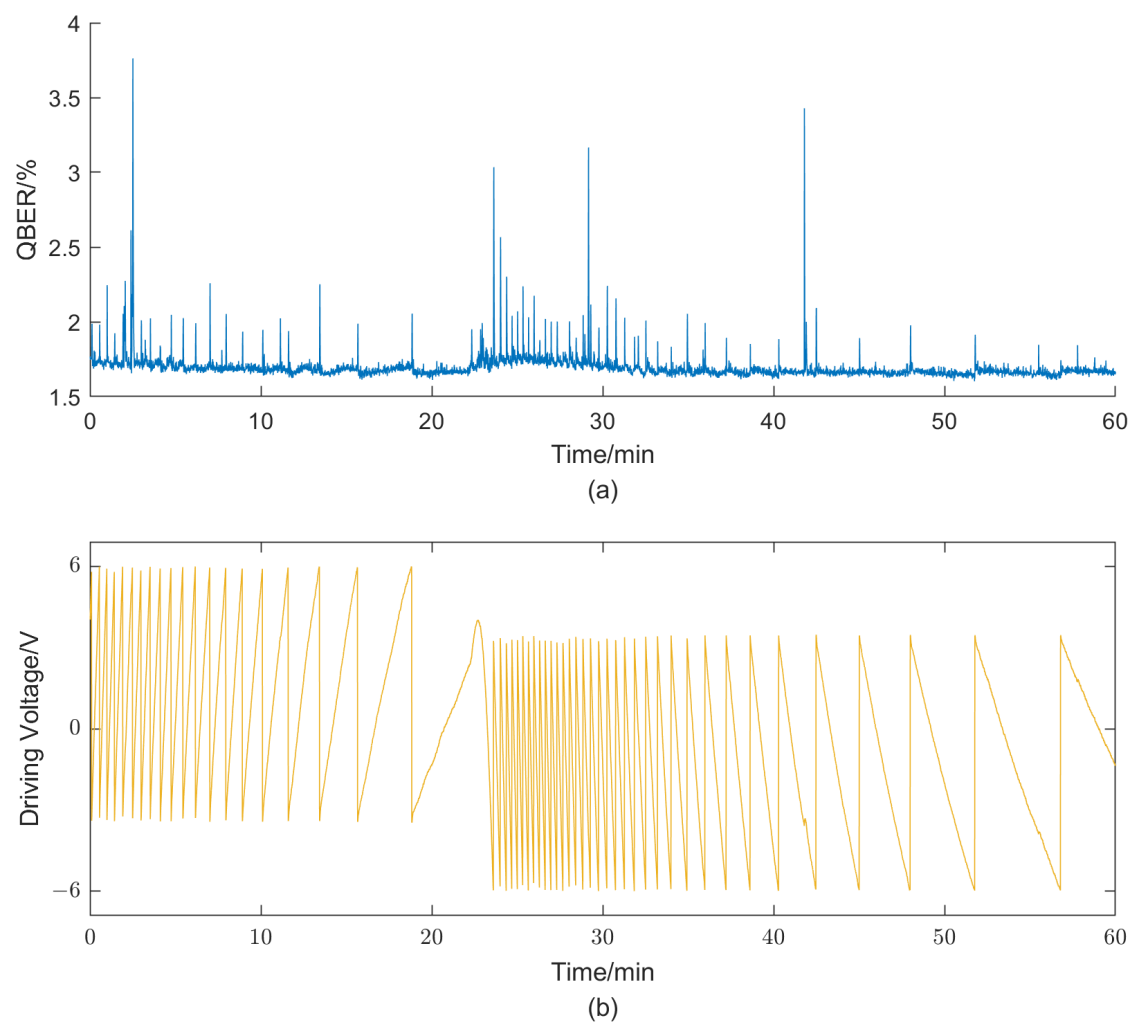
Obtained QBER (**a**) and driving voltage of the phase modulator (**b**) with a feedback compensation frequency of 1 s per iteration.

**Figure 5:**
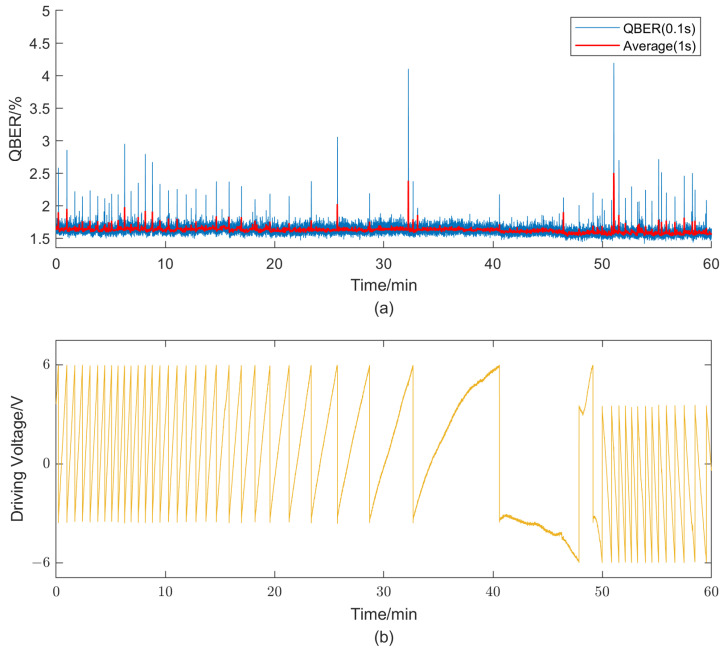
Obtained QBER (**a**) and driving voltage of the phase modulator (**b**) with a feedback compensation frequency of 0.1 s per iteration.

To better simulate the complex field environments, a hot air fan is used to blow the interferometer for about half an hour, followed by a subsequent cooling treatment. Figure 4 depicts the experimental results obtained from running the system for 1 h. The results demonstrate a high overall stability of the system, with an average QBER value of 1.69% and a standard deviation of 0.0813%. However, a noticeable difference is still observed compared to the previously measured value $e_{0}$. Considering the rapid velocity of phase shift, in order to mitigate this discrepancy, we increase the frequency of feedback compensation from 1 s to 0.1 s per iteration. Figure 5 illustrates the test results under similar experimental conditions as before, showing a distinct decrease in QBER. The average value and standard deviation reached 1.62% and 0.0687%, respectively. Moreover, the standard deviation decreased to 0.0425% when performing statistical calculations at a rate of every 1 s. This result validates the effectiveness of improving the feedback speed in enhancing the overall system performance. It should be noted that both sets of experimental results exhibit noticeable periodic fluctuations in QBER, primarily occurring during full-cycle voltage adjustments due to limited driving voltage. This is mainly attributed to insufficient modulation bandwidth of the modulation electrode and its temperature-dependent half-wave voltage drift. The limited modulation bandwidth results in a longer time required for the modulator to reach the target value. During this period, the optical pulses passing through the modulator are subjected to inaccurate modulation. Additionally, the inaccuracy in the half-wave voltage directly leads to errors in the modulation voltage. Both of these situations can cause an instantaneous increase in the QBER.

**Figure 6:**
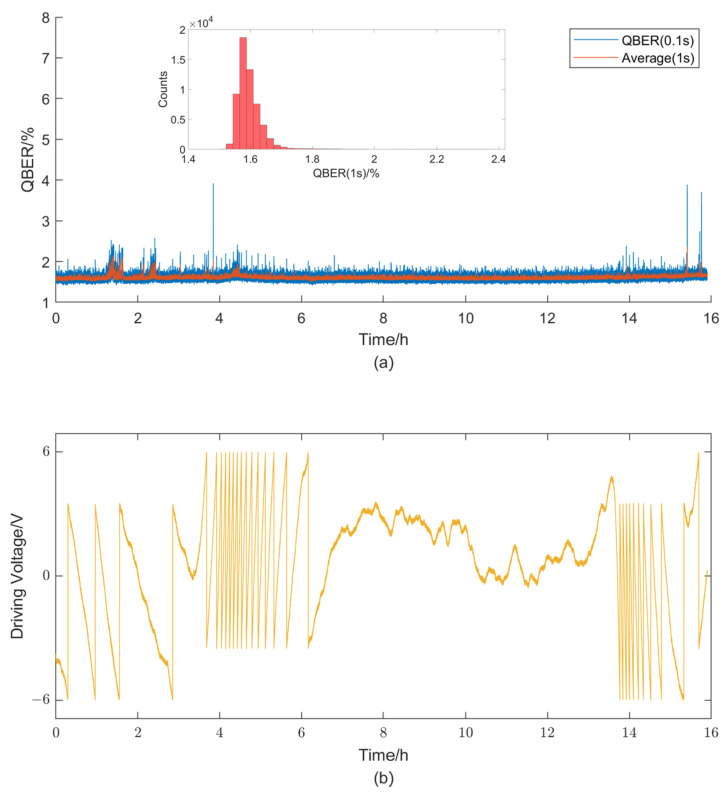
QBER, its statistical distribution (**a**), and driving voltage of the phase modulator (**b**) with a feedback compensation frequency of 0.1 s per iteration for 16 h.

To further validate the long-term stability of the system, we conduct an extended stability test lasting nearly 16 h with a feedback compensation frequency of 0.1 s per iteration. Figure 6 presents the QBER, its statistical distribution, and the variations in feedback driving voltage. The obtained QBER achieves an average value of 1.60% and a standard deviation of 0.0583%.

## 4. Conclusions

In this paper, we propose a real-time phase drift compensation scheme for phase-coding quantum communication systems, in which only existing data from the quantum communication process is used to establish a stable closed-loop control subsystem for phase tracking without any additional transmission efficiency reduction or information exchange process. Our scheme is applied into an FSMI-based phase-coding quantum communication system, leading to a stable and continuous operation, even with a complex environmental disturbance. Note that, in our long-term stability experiment, we obtain an average QBER value of 1.60%, which is approximately equal to $e_{0}=1.59\%$, and the effectiveness of our scheme has been effectively validated. Additionally, it can be observed from the experimental results that increasing the statistical frequency can effectively improve system performance. However, this also results in a reduced number of statistical samples, leading to larger statistical errors. Therefore, a careful balance is needed in this regard, especially when the repetition rate is lower, which results in relatively smaller counts and larger fluctuations. Another point that needs to be mentioned is that, during the phase tracking process, there is still a possibility of incorrect modulation direction due to sudden phase changes or statistical fluctuations when the phase tracking driving voltage operates near the target value. Consequently, the incorrect modulation direction results in a higher QBER, which is a clear indication of modulation direction errors and can be quickly corrected through subsequent compensation, leading to a relatively stable state. The stability of the experimental results also verifies this point.

## Data Availability

No new data were created or analyzed in this study. Data sharing is not applicable to this article.
